# Hurricane-induced power outage risk under climate change is primarily driven by the uncertainty in projections of future hurricane frequency

**DOI:** 10.1038/s41598-020-72207-z

**Published:** 2020-09-17

**Authors:** Negin Alemazkoor, Benjamin Rachunok, Daniel R Chavas, Andrea Staid, Arghavan Louhghalam, Roshanak Nateghi, Mazdak Tootkaboni

**Affiliations:** 1 School of Industrial Engineering, Purdue University, West Lafayette, IN USA; 2 Department of Earth, Atmospheric, and Planetary Sciences, Purdue University, West Lafayette, IN USA; 3 Department of Discrete Math and Optimization, Sandia National Laboratories, Albuquerque, NM USA; 4 Department of Civil and Environmental Engineering, University of Massachusetts Dartmouth, Dartmouth, MA USA; 5 Environmental and Ecological Engineering, Purdue University, West Lafayette, IN USA; 6 Department of Engineering Systems and Environment, University of Virginia, Charlottesville, VA USA

**Keywords:** Natural hazards, Energy infrastructure, Climate change

## Abstract

Nine in ten major outages in the US have been caused by hurricanes. Long-term outage risk is a function of climate change-triggered shifts in hurricane frequency and intensity; yet projections of both remain highly uncertain. However, outage risk models do not account for the epistemic uncertainties in physics-based hurricane projections under climate change, largely due to the extreme computational complexity. Instead they use simple probabilistic assumptions to model such uncertainties. Here, we propose a transparent and efficient framework to, for the first time, bridge the physics-based hurricane projections and intricate outage risk models. We find that uncertainty in projections of the frequency of weaker storms explains over 95% of the uncertainty in outage projections; thus, reducing this uncertainty will greatly improve outage risk management. We also show that the expected annual fraction of affected customers exhibits large variances, warranting the adoption of robust resilience investment strategies and climate-informed regulatory frameworks.

## Introduction

Energy infrastructure is the backbone of our digital economy as well as societal health and safety, and uninterrupted access to electricity is essential for sustaining modern life^2^. Hurricanes are the leading cause of major power outages^26^ and have been responsible for over $900 billion in damage and thousands of fatalities in the US over the last 40 years^30,36^. In 2012, for example, Hurricane Sandy left over 8.5 million customers in the U.S. without power and caused over $7 billion in economic loss and 225 fatalities^35^.

In an effort to minimize the impact of hurricane-induced power outages, several research studies have developed forecast models to provide estimates of the extent of power outages prior to a storm landfall^7,29,31,33,34,37^. Such short-term forecasts allow utilities to improve their preparation, response and recovery efforts. They also allow emergency management and response agencies to better plan population evacuations and locate emergency shelters^12,27,32^. However, long term resilience investment decisions in electric power systems require the ability to credibly characterize long-term power outage risks, warranting the inclusion of climate change as a force affecting hurricane activity^6^.

There exists a significant body of literature on the relationship between climate change and hurricane activity, with most studies reporting a decrease in storm frequency due to climate change^20,48,49,53^. However, a non-trivial number of studies project an increase in frequency of hurricanes under similar future climate scenarios^3,10,56^. Different choices of climate models, initial parametrization of the models^21^, and spatial resolution of the models^52^ are among the reasons that cause such variability in projections of future hurricane activity. There is also much uncertainty about the projected future hurricane intensity under climate change. While there is consensus about an increase in future storm intensity, the extent of the projected increase varies among different studies^20^ ranging from 2 to 14%^14,20,21,54^. Importantly, regional predictions of hurricane activity under climate change depend on the pattern of warming and so may be fundamentally limited on these long timescales^20,22^. Thus, it is not clear that science will be able to reduce these epistemic uncertainties in the foreseeable future. Moreover, potential economic damages are known to increase with storm frequency and intensity^19,39^, including at the extremes^4^.

While many studies have investigated the impacts of climate change on electricity demand and supply^28,38,41^, very few have examined climate-change driven hurricane risk to electric power distribution systems^12,47^ which are most vulnerable to sever weather and climate events^16^. Resilient electric power infrastructure planning in hurricane prone regions hinges on the ability to systematically characterize and efficiently propagate the uncertainties in hurricane activity under climate change. However, existing approaches rely on computationally expensive methods, which limits their potential for adoption by local and regional planners as well as regulating agencies. Moreover, the physics-based projections of hurricane activity under climate change are not integrated into the existing hurricane outage risk models under climate change. Instead, simple probabilistic assumptions are made to perturb hurricane activity.

We address these fundamental gaps by proposing a transparent and efficient approach to, for the first time, link the knowledge provided by physics-based projections of hurricane activity under climate change with electric power distribution infrastructure risk models, providing a pathway for better integration of climate physics within intricate engineering risk models. We do this by coupling the latest synthesis of physics-based climate change projections with a validated power outage forecast model to predict the impact of future hurricane activity on coastal power distribution systems and then attribute the uncertainty in power outage risk to epistemic uncertainty in future frequency and intensity of hurricanes. Specifically, we utilize results from a recently-published study of projections of hurricane activity^20^ to quantify the uncertainty in these projections and fit parameterized distributions to future intensity and frequency of hurricanes. This allows us to efficiently propagate the uncertainty in projections through a validated hurricane-induced outage model^13,47^ in order to directly link changes in hurricane activity to outage risk. Particularly, we seek to answer two fundamental questions: (a) is long-term power outage risk driven primarily by climate change-triggered shifts in intensity or frequency?, and (b) what is the distribution of the expected annual outages due to climate change triggered shifts in hurricane frequency and intensity?

We find that hurricane-induced power outage risk under climate change is predominantly driven by the uncertainty in future frequency of hurricanes. Precisely, uncertainty in future projections of the frequency of hurricanes explains over 95% of the total uncertainty in future outage projections. We also show that the expected annual fraction of customers affected by power outage exhibit rather large variances. Given the multi-decadal timescales of power infrastructure planning, this wide range of possible future outcomes highlights the urgent need for a more robust approach for setting and enforcing reliability standards toward effective management of the power system under climate change.

## Projected outage risk

To characterize long-term power outage risk, we first create a baseline scenario to represent present-day hurricane impacts on power distribution systems. The baseline scenario is generated by simulating 2000 years of storm-induced outages, using historical data and a validated outage forecast model^13^. To examine impacts under climate change then, our model takes as input three factors that define changes in hurricane activity and whose distributions are provided in a recent synthesis work^20^: the overall frequency factor, $f_{fre}$, the frequency factor for intense storms, $f^{sev}_{fre}$, and the intensity factor, $f_{int}$. Distinguishing between overall vs. intense storm frequency allows for shifts within the distribution toward higher-intensity storms. These factors represent fractional increases or decreases in their respective components in a future climate state. They are set to one under the baseline scenario and take uncertain values according to climate change projections. Their distributions may be directly estimated from the projections of hurricane activity under climate change. Here, we fit parameterized distributions to the data from the latest synthesis of physics-based climate change projections by Knutson et al.^20^ (see “Methods” for details). We then leverage an efficient methodology for stochastic computations^59^ to propagate the uncertainty in hurricane activity through the outage forecast model to evaluate future power outage risk and its range of uncertainties. Finally, we leverage global sensitivity analysis^44^ to investigate whether the uncertainty in long-term power outage risk is driven primarily by the uncertainty in future intensity or frequency (“Methods”). Our proposed framework is computationally efficient, and it offers a simple and transparent way for quantitative attribution of infrastructure impact risk to key parameters governing changes in hurricane activity. These key advantages render the framework ideal for decision-support tools under climate change and provide a path for integrating climate knowledge within infrastructure impact models to better inform planners, regulators, and policymakers.

**Figure 1:**
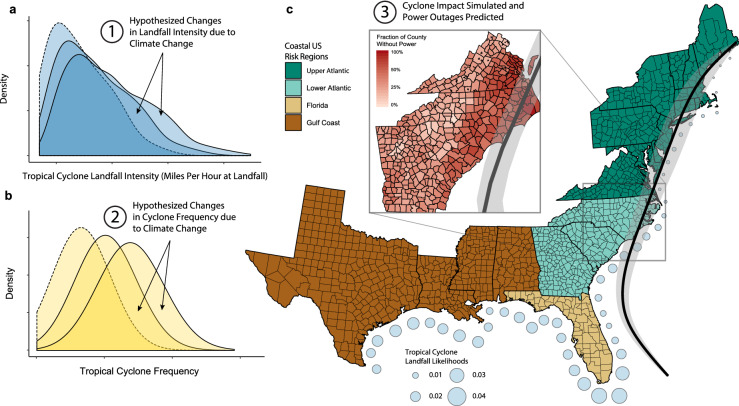
The schematic of the key sources of uncertainty in hurricane activity (**a**,**b**) and a typical outage map (inset) for a given simulated hurricane in the region under study (**c**). The intensity (**a**) and frequency (**b**) of landfalling hurricanes is perturbed from the baseline (dotted line) scenario in line with climate change projections to create future (solid line) scenarios. These scenarios simulate climate-perturbed distributions of tropical cyclone intensity and frequency which are used to simulate storm landfall along the Gulf and Atlantic coasts of the US (**c**). Note the distributions in (**a**,**b**) and the outage map depicted in the inset in (**c**) do not depict actual values. Plots a-b were created in R (v 3.2.1; https://www.r-project.org/)^40^ using the ggplot2 (v 3.3.0; https://ggplot2.tidyverse.org/)^57^. Plot (**c**) was created in R (v 3.2.1; https://www.r-project.org/)^40^ using the packages ggplot2 (v 3.3.0; https://ggplot2.tidyverse.org/)^57^ and usmap (v 0.5.0; https://github.com/pdil/usmap)^8^. Map shapefiles were from usmap and the US Census Bureau^51^.

**Figure 2:**
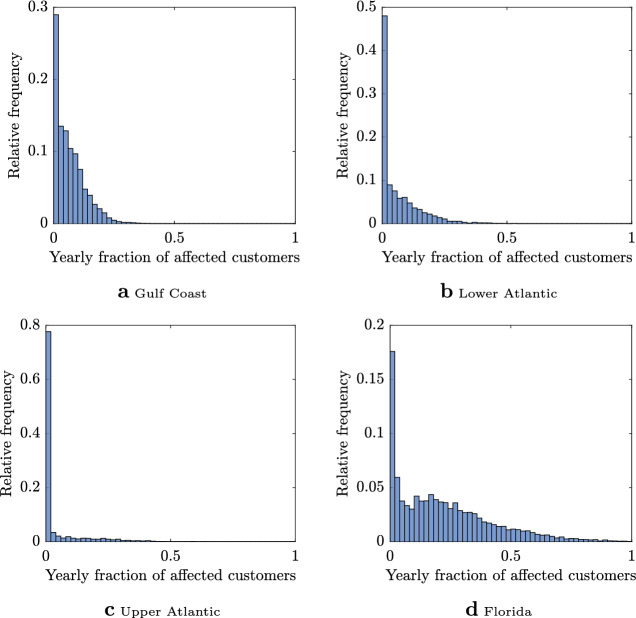
Distributions of the yearly fractions of customers without power in each coastal region under baseline scenario. Plots (**a**–**d**) were created in MATLAB R2016a.

To simulate the 2,000 years of storm-induced power outages under the baseline scenario, the frequency and intensity of hurricanes are sampled from historical distributions; i.e., the dashed line distributions in Fig. 1a,b. Landfall location is drawn from the historical record aggregated to 50km stretches of the coastline as shown by the circles in Fig. 1c. Hurricane tracks and wind fields (see thick black line with the gray band in Fig. 1c) are also modeled using historical data and then fed to a validated power outage forecast model^13,47^ (““Methods”) to estimate the outage risk in terms of the fraction of the population that loses power in each simulated storm (colored map in Fig. 1c). The fractions are finally aggregated across four distinct regions: the Gulf, upper Atlantic, lower Atlantic and Florida. Figure 2 depicts the distributions of the yearly fractions of customers affected by power outage in these four US regions for the baseline scenario. Based on these distributions, we calculate baseline statistics critical for decision making: the expected value, $\bar{F}$, and the 95th percentile, $F_{95}$, (see Supplementary Information for the latter) of the yearly fraction of affected population.

**Figure 3:**
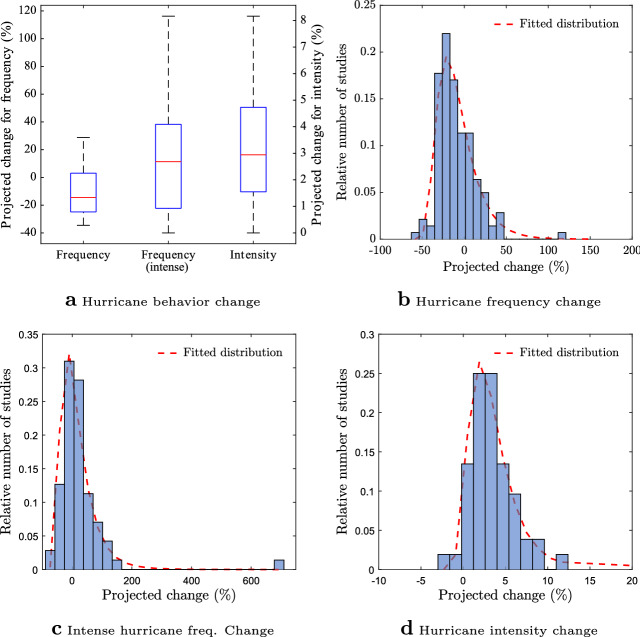
Projected changes in hurricane frequency and intensity for the North Atlantic basin. (**a**) Box plots with interquartile ranges used to define the frequency factor, $f_{fre}$, the frequency factor for intense storms, $f^{sev}_{fre}$, and the intensity factor, $f_{int}$, (**b**–**d**) Histograms pertaining to the data for each factor taken from Figures 1–3 of Knutson et al.^20^ and the fitted shifted log-normal distributions. Plots (**a**–**d**) were created in MATLAB R2016a.

To perturb hurricane activity under climate change, we assign probability distributions to the frequency factor, the frequency factor for very severe storms, and the intensity factor, $f_{fre}$, $f_{fre}^{sev}$ and $f_{int}$, based on the recently-published synthesis of future hurricane projections for a 2*K* global mean temperature increase by Knutson *et al.*^20^. Figure 3a–c, generated directly from the raw data (Knutson, pers. comm.), depict the variability in these factors for the North Atlantic basin. It can be seen that hurricane frequency is mostly projected to decrease, but the frequency of very intense storms, i.e., category 4 and 5, is expected to increase. Considering the longer upper tail of the distributions, we model each factor using shifted log-normal distributions with the same interquartile ranges as those reported in Knutson et al.^20^.

**Figure 4:**
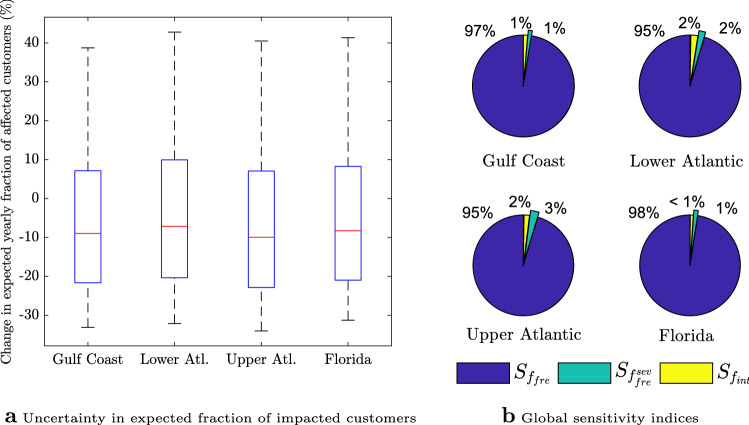
Uncertainty quantification and sensitivity analysis of the expected yearly fraction of impacted customers under climate change. (**a**) Box plots of the change (w.r.t baseline scenario) in the expected yearly fraction of affected customers, $\bar{F}$, for the four US regions. The figure shows the median, the interquartile range, the 5th and 95th percentiles of the change in the expected yearly fraction of customers that experience hurricane-induced power outage, given the uncertainty in the impact of climate change on frequency and intensity of storms, (**b**) Sobol’ global sensitivity indices characterizing the contribution of factors $f_{fre}$, $f_{fre}^{sev}$ and $f_{int}$ to the variance of the yearly fraction of costumers without power, $\bar{F}$. The variability in hurricane frequency is the main contributor to the variance of $\bar{F}$ in all four regions under consideration. Plots a-b were created in MATLAB R2016a.

Given the probability distributions of the frequency factor, the frequency factor for intense storms, and the intensity factor, we obtain the probability distribution of expected yearly fraction of affected customers through building a Polynomial Chaos surrogate^11,59^ (“Methods”). Figure 4a shows the median, the interquartile range, and the 5th and 95th percentiles for the change in the expected yearly fraction of affected customers in the four considered regions. It is found that (see Supplementary Information) there is about 60–65% chance that the expected fraction of customers without power will decrease under climate change, in all regions. Importantly, the changes in the expected yearly fraction of affected customers for all regions exhibit rather large variances, with 90% confidence intervals that span a wide range from large (more than 30%) decreases to large (about 40%) increases. This large variability in the expected yearly fractions of impacted customers makes power system resilience planning and management especially challenging. Can we understand what causes such a large uncertainty?

To answer this question, we next investigate whether this long-term power outage risk is driven primarily by the uncertainty in future intensity or frequency. This is done through a global sensitivity analysis using Sobol’ sensitively indices^45^. The Sobol’ indices, illustrated in Fig. 4b, represent portions of variance of expected yearly impacted customers attributed to the uncertainty in frequency factor, the frequency factor for very severe storms, and the intensity factor. It can be seen that, in all four regions, more than 95% of variability in the expected yearly fraction of customers that experience power outage is caused by the uncertainty in frequency of non-intense tropical storms. Additionally, in all regions, more than 97% of observed variability in Fig. 4a is due to the uncertainty in future frequency of storms, i.e., both non-intense and intense storms. On the other hand, uncertainty in future intensity of storms barely contributes to the variability in the expected fraction of impacted costumers.

The non-significant contribution of uncertainty in the intensity of storms to power outage risk can be explained by the small variance of the change in future storm intensity. In fact, it is evident in Fig. 3a that the range of projected change in hurricane intensity in North Atlantic basin is relatively small. On the other hand, that same figure shows that the variability in projected climate change-triggered changes in the frequency of non-intense and intense storms is large. Yet, the variability in projected frequency of intense storms contributes to less than 3% of variability in power outage risk in all regions. This can be explained by the small frequency of intense storms for the baseline scenario with an average of 0.12 per year compared to 4.19 for non-intense storms. An extreme scenario of a 100% increase in the frequency of future storms, for instance, translates to average frequencies of 0.24 and 8.38 storms per year for intense and non-intense storms, respectively. Consequently, it is mainly the variability in future *frequency of non-intense hurricanes* which drives the uncertainty in the expected faction of affected customers. This suggests that more precise projections of the frequency of non-intense hurricanes are crucial to arrive at significantly improved predictions of hurricane-induced power outage risk under climate change.

## Discussion

Hurricanes are among the most costly and devastating natural hazards, wreaking havoc on the built environment each year. Climate change is projected to shift hurricane activity. However, there is little consensus on the degree of shifts in hurricane activity among different climate models and it is not clear whether science will be able to reduce this epistemic uncertainty anytime soon. These uncertainties may render the current long-term infrastructure resilience investments sub-optimal under climate change. Moreover, existing approaches for characterizing uncertainty in projected risk are computationally expensive “black boxes” that are not practical for use in long-term planning. In addition, there is often a disconnect between the physics-based climate projections and infrastructure risk models. We propose a novel and yet computationally cheap framework to propagate the uncertainties in climate projections to infrastructure risk models and to characterize the primary driver of uncertainty in hurricane-induced power outage risk. Specifically, we propagate the uncertainty in projection of future hurricane activity defined by a recently-published synthesis study^20^ to power outage risk under climate change using a validated state-of-the-art power outage model. While our uncertainty-informed analysis reveals a modest chance of decrease in the expected yearly fraction of affected customers under climate change, we also find a very wide range of outcomes, from more than 30% decrease to about 40% increase, which translates to significant risk for long-term power grid planning. Specifically, this variability needs to be accounted for in power distribution systems contingency planning in terms of both emergency operations and systems hardening. It is also of critical importance for utility regulatory commissions that tend to set reliability standards based on historical system performances^35^.

We exploit the simplicity of our framework to demonstrate the uncertainty in long-term service disruptions in the power distribution systems is primarily driven by the uncertainty in projection of frequency of non-intense hurricanes. Our proposed framework can be used by electric utility planners and policymakers to make uncertainty-informed resilient investment decisions. It can also provide insights to climate scientists on the practical implications of uncertainty in climate change projections for power systems management. In particular, the results suggest that efforts that can reduce the uncertainty in predictions of future frequency of hurricanes of all intensities more accurately on the basin scale, or ideally the regional scale, will substantially improve the predictions of hurricane-induced power outage risks under climate change.

We finally note that the study presented here can be extended to account for potential changes in distribution of landfall locations for a more comprehensive analysis of power outage risk under climate change^24^. Additionally, the statistical relationships in the outage model used here can be coupled with a simulation model to include grid-hardening measures such as undergrounding power lines. By formulating outage models based on alternative grid designs or topologies, our framework would allow for considering technological changes, such as an increased adoption of renewable energy or distributed generation, in evaluating long term power outage risks.

## Methods

### Baseline scenario: historical data and the simulation framework

The historical data and simulation framework used to generate the baseline scenario in this study are based on the work by Staid et al.^47^; below we provide a summary, and refer the reader to the original text for details.

#### Storm seeding

We rely on the historical record of hurricanes making landfall in the continental United States to determine the baseline behavior of hurricanes. Based on storms occurring between 1851 and 2018, we fit a Poisson distribution to annual frequency of storms making landfall and build empirical distributions for the location of landfall and the storm intensity using the HURDAT2 database^25,50^. For each replicated year, a realization is drawn from the distribution of annual frequency to determine the number of storms occurring. For each simulated storm within a given year, sample realizations are drawn from the distribution of historical landfall locations and distribution of maximum windspeed at landfall. These samples determine where the storm makes landfall and how strong it is when it does.

#### Storm track model

For each simulated storm, we create a track using a Random Forest model trained on historical storm movement in the same region. For example, for landfall locations in the Gulf of Mexico we rely on historical storms along the Gulf Coast to train the Random Forest model. This ensures that the storm movement follows realistic weather patterns. The track is created concurrently with simulated storm decay. For each six-hour time step following landfall, the maximum wind speed decays according to the model proposed by Kaplan and Demaria^18^. This proceeds until the maximum wind speed falls below the threshold for tropical storm classification (1-min sustained winds of 34 knots).

#### Wind field model

With the storm track and forward moving speed fully specified, we then employ a wind field model to calculate the expected distribution of winds for the areas exposed to the storm. To characterize the wind field in this study, we adopt a model proposed by Willoughby et al.^58^ and use the stormwindmodel R package^1^ to calculate the storm radius, the maximum 3-s gust wind, and the duration of winds above 20 m/s for the centroid of each census tract within the storm’s radius.

#### Power outage model

Finally, we use wind parameters calculated via the wind field model as input to a statistical power outage prediction model developed by Guikema et al.^13^, The model has been trained and validated on historical hurricane-induced power outage events to represent the relationship between wind characteristics and the fraction of customers expected to lose power from a given storm.

### Climate-informed projection

To study the impact of climate change on $\bar{F}$, we introduce three multiplicative factors: the frequency factor, $f_{fre}$, the frequency factor for very severe storms, $f^{sev}_{fre}$, and the intensity factor, $f_{int}$. Specifically, to account for the changes in the frequency of storms, we multiply the mean of the Poisson distribution, that is fitted to the historical data and is used to sample the number of storms in each replicated year, by the frequency factor. Since several studies have suggested that the change in frequency of very intense storms shows a different pattern^20^, we assign a specific multiplicative factor, $f^{sev}_{fre}$, to the mean of Poisson distribution for frequency of very intense storms. Lastly, to account for the changes in intensity of storms, we multiply the randomly sampled maximum wind speed for each hurricane by the intensity factor, $f_{int}$. Note that $f_{fre}$, $f^{sev}_{fre}$, and $f_{int}$ are equal to one under baseline scenario and their values under climate change are uncertain. Their uncertainty are evident in Fig. 3, which is reprinted from a synthesis study by knutson *et al.*^20^. Considering the heavy tails of distributions for North Atlantic basin, we assign shifted log-normal distributions with the same interquartile ranges depicted in Fig. 3 to represent the variability in $f_{fre}$, $f_{fre}^{sev}$ and $f_{int}$. We then sample from these log-normal distributions to draw realizations for the frequency factor, the frequency factor for very severe storms, and the intensity factor for each simulation year under climate change. Note also that we do not consider the impact of climate change on storm sizes in our analysis. We do this given that the storm size is not expected to change significantly with climate change^23^, in line with recent theoretical advances in our understanding of storm size^5^.

#### Polynomial chaos surrogate and sensitivity analysis

To facilitate the uncertainty quantification, we approximate $\bar{F}$ as series expansions in orthonormal Hermite polynomials (Polynomial Chaos) of $f_{fre}$, $f_{fre}^{sev}$, and $f_{int}$. We use regression to approximate the coefficients of the series expansion and select the polynomial order and number of training samples required for surrogate construction through convergence analysis. Interested readers are referred to^11,17,55,59^ for more details and theoretical background on Polynomial Chaos surrogates and their computational efficiency as uncertainty quantification engines.

We use one of the most widely used global sensitivity analysis approaches, namely Sobol’ method^43,45^. In this approach, the variance of system’s response is decomposed to summation of contributions from different inputs to the model and their interaction. The sensitivity of a particular response (say the expected fraction of costumers without power) to different inputs (say the frequency factors) then manifests itself in their associated Sobol’ indices. More specifically, a square-integrable function $u(\varvec{\Xi })$ is expressed as a sum of elementary basis functions^15^:

1 $$\begin{aligned} u(\varvec{\Xi })= u_0 + \sum _{j=1}^{d}u_j(\Xi _j)+\sum _{j<i}^{d}u_{ij}(\Xi _j,\Xi _i ) +\cdots + u_{12\cdots d}(\varvec{\Xi }), \end{aligned}$$

where *d* is the dimensionality of the input, $\varvec{\Xi }$, and $u_0$ is a constant. It was shown by Sobol^46^ that the above decomposition is unique if the basis functions in the expansion are orthogonal. Accordingly, a functional decomposition of the variance is available^9^:

2 $$\begin{aligned} \text {Var}(u(\varvec{\Xi }))= \sum _{j=1}^{d}D_j+ \sum _{j<i}^{d}D_{ij} +\cdots + D_{12\cdots d}, \end{aligned}$$

where $D_i= \text {Var} [\mathbb {E}(u(\varvec{\Xi })| \Xi _i)]$, $D_{ij} =\text {Var} [\mathbb {E}(u(\varvec{\Xi })| \Xi _i,\Xi _j )]- D_i-D_j$ and so on for higher order interactions with $\mathbb {E}$ the operation of mathematical expectation. Finally, the variance-based sensitivity indices, the so-called Sobol’ indices, read

3 $$\begin{aligned} S_i=\frac{D_i}{\text {Var}(u(\varvec{\Xi }))}, \ \ S_{ij} =\frac{D_{ij}}{\text {Var}(u(\varvec{\Xi }))}, \ \cdots \ . \end{aligned}$$

It is readily understood that the Sobol’ indices also satisfy

4 $$\begin{aligned} \sum _{j=1}^{d}S_j+ \sum _{j<i}^{d}S_{ij}+ \cdots + S_{12\cdots d}=1. \end{aligned}$$

The Sobol’ indices, therefore, specify the contribution to the total variance, $\text {Var}(u(\varvec{\Xi }))$, from given inputs or input combinations. Monte Carlo sampling based approaches can also be used to evaluate the Sobol’ indices^42^. However, these approaches can be computationally costly due to the small convergence rate of Monte Carlo sampling particularly in the case of this study when the sought statistic is that of a expected value which itself is based on upwards of tens of hundreds of “simulated years”. Alternatively, Sobol’ indices can be calculated with minimal computational cost when $u(\varvec{\Xi })$ is approximated as series expansion of orthonoromal polynomials. For example, $D_i$ in Eq. (3), can be readily calculated as sum of squares of the coefficients associated with the subset of the polynomial basis functions which are functions of $\Xi _i$ only. Similarly, $\text {Var}(u(\varvec{\Xi }))$ can be calculated as summation of squared coefficients of all polynomial basis functions with order equal to or greater than one.

## Supplementary information

Supplementary information

## Data Availability

The simulation code and data are available upon request.
