# Supplementary material for: Hurricane-induced power outage risk under climate change is primarily driven by the uncertainty in projections of future hurricane frequency
Source: Sci Rep. 2020 Sep 17;10:15270. doi: 10.1038/s41598-020-72207-z (PMC7499252; doi:10.1038/s41598-020-72207-z)
Supplement: Supplementary file 1 — Supplementary information [file 41598_2020_72207_MOESM1_ESM.pdf]

Hurricane-induced power outage risk under climate change is primarily driven by the uncertainty in projections of future hurricane frequency

Supplementary Information

N. Alemazkoor[1,4], B. Rachunok[1], D.R. Chavas[2], A. Staid[3], A. Louhghalam[4], R. Nateghi[1,5], Mazdak Tootkaboni[4]

[1]School of Industrial Engineering, Purdue University, West Lafayette, IN
[2] Department of Earth, Atmospheric, and Planetary Sciences, Purdue University, West Lafayette, IN
[3]Department of Discrete Math & Optimization, Sandia National Laboratories, Albuquerque, NM
[4]Department of Civil and Environmental Engineering, University of Massachusetts Dartmouth, MA
[5]Environmental and Ecological Engineering, Purdue University, West Lafayette, IN

Supplementary Note 1: Convergence Analysis

As mentioned in the main body of manuscript, the baseline scenario is generated by simulating 2000 years of storm-induced outages. The number of realizations (simulated years) is chosen based on a convergence analysis to ensure important statistics of yearly affected customers are calculated accurately. Figure 1 shows the evaluated mean and variance of yearly fraction of customers affected versus the number of simulated years. It can be seen that running the simulation (storm seeding, storm track and wind field modeling, and power outage modeling) for 2000 years is certainly enough to reach convergence.

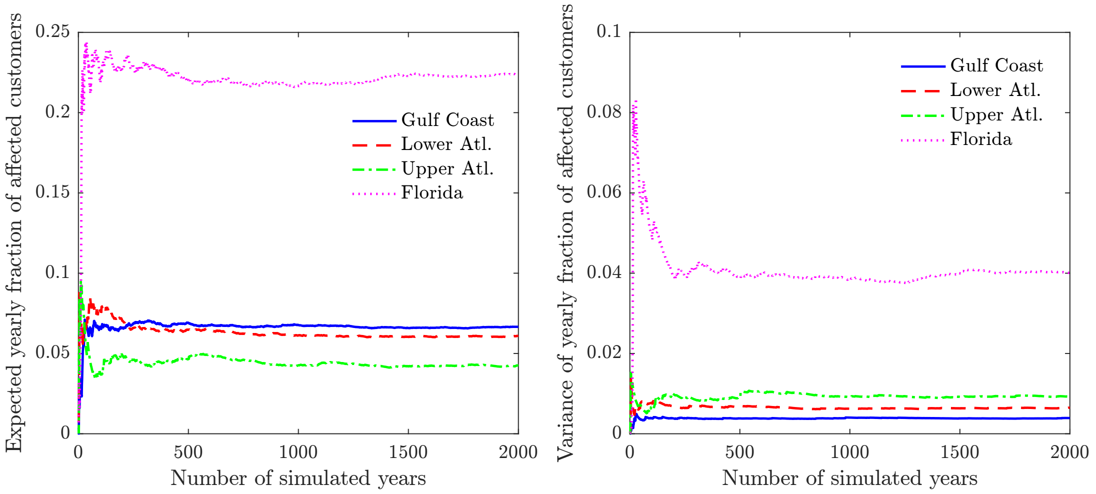

a Convergence of mean **b** Convergence of Variance

Figure 1: Convergence analysis for the mean and variance of the yearly fraction of affected customers under baseline scenario for the four regions. Plots a-b were created in MATLAB R2016a.

Supplementary Note 2: Distributions of Expected Yearly Fraction of Affected Customers

The change in the expected yearly fraction of affected customers, $\bar{F}$, with respect to baseline scenario is depicted in Figure 4a in the main body of the manuscript. Figure 2 demonstrates the distributions of expected yearly fraction of customers affected by hurricane-induced power outage for the four regions under consideration obtained using the Polynomial Chaos surrogate (Methods). Red crosses show the expected yearly fraction of affected customers under the baseline scenario. A close examination of these distributions reveals the chance that the expected fraction of affected customers will decrease is about 60-65%. However, there is a fairly large chance (about 35%-40%) that the expected value of the fraction of customers affected increases. Furthermore, given the large uncertainty in the impact of climate change on hurricanes activity, the possible changes in $\bar{F}$ span a wide range from more than 30% decrease to about 40% increase, a level of variability that needs to be taken into account in contingency planning from both the systems hardening and emergency operations perspectives.

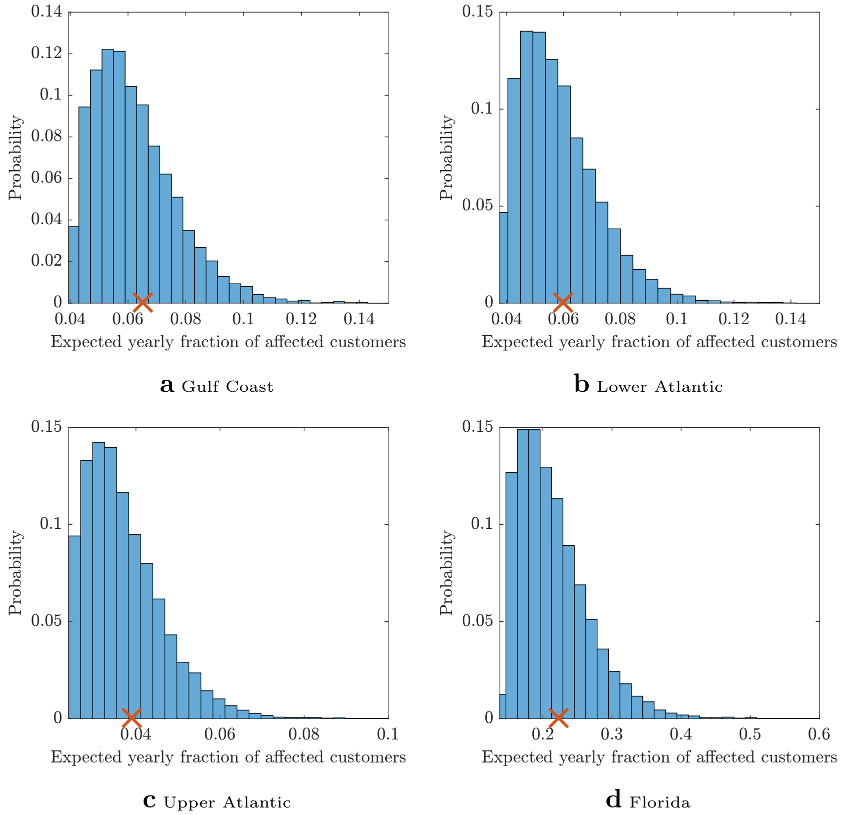

Figure 2: Distributions of expected yearly fraction of affected customers, $\bar{F}$, for each coastal region under climate change. Red crosses show the expected yearly fraction of affected customers under the baseline scenario. Plots a-d were created in MATLAB R2016a.

Supplementary Note 3: Impact of Climate Change on the Tail of Power Outage Distribution

In the main body of the manuscript, we proposed a framework to examine the expected yearly fraction of costumers that experience storm-induced power outage, $\bar{F}$, and its projected variability under climate-change induced uncertainty. It is worthwhile to utilize the same "machinery" to investigate how climate change impacts the tail of power outage distribution, e.g. the 95th percentile of yearly fraction of costumers affected by power outage, F_{95}. To this end, we regenerate the results in Figures 4a and 4b with F_{95} now the output of interest. The results in Figure 3a show the median, the interquartile range, and the 5th and 95th percentiles of the change

in the 95th percentile of yearly fraction of customers that experience tropical cyclone-induced power outages for the four considered coastal areas. It is observed that for all four coastal areas, there is a fair chance that the 95th percentile of yearly fraction of impacted customers by power outage will decrease under climate change. As an example, given the uncertainty in estimated future frequency and intensity of hurricanes, there is a 65% probability that the 95th percentile of yearly fraction of customers impacted by power outage will decrease in Gulf Coast. The sensitivity of the tail of yearly affected costumers to the multiplicative factors f_{fre}, f_{fre}^{sev}, and f_{int} for four US regions are depicted in Figure 3b in the form of Sobol' indices. Similar to the mean fraction of customers that experience power outage, more than 90% of variability in the 95th percentile of yearly fraction of impacted customers is attributed to the uncertainty in frequency of non-intense tropical storms and more than 95% of variability is due to the combined uncertainty in future frequency of storms, both non-intense and intense.

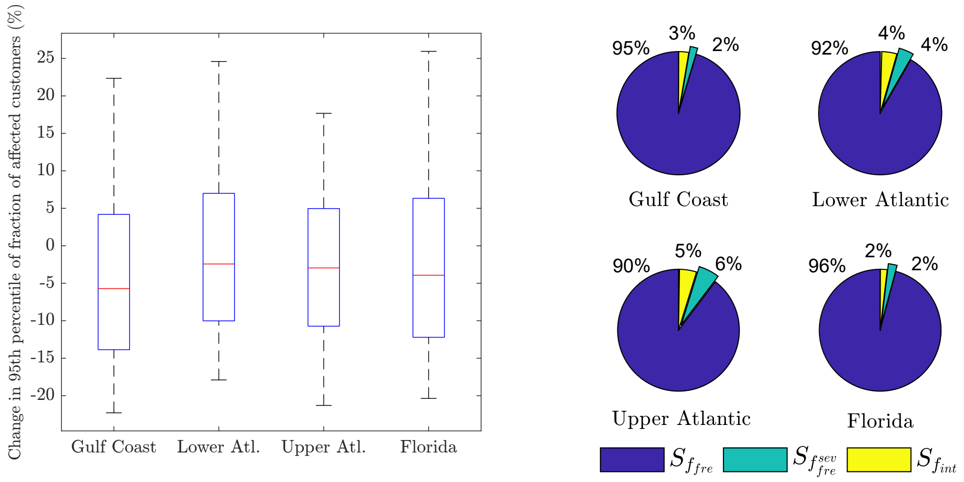

a Uncertainty in 95th percentile of yearly fraction of impacted customers

b Global sensitivity indices

Figure 3: Uncertainty quantification and sensitivity analysis of the 95th percentile of yearly fraction of impacted customers under climate change. **(a)** Box plots of the change (w.r.t. baseline scenario) in the 95the percentile of yearly fraction of affected customers, F_{95}, for the four US regions. The figure shows the median, the interquartile range, the 5th and 95th percentiles of the change in the 95the percentile of yearly fraction of customers that experience hurricane-induced power outage, given the uncertainty in the impact of climate change on frequency and intensity of storms, **(b)** Sobol' global sensitivity indices characterizing the contribution of factors f_{fre}, f_{fre}^{sev} and f_{int} to the variance of 95th percentile of yearly fraction of costumers without power, F_{95}. The variability in hurricane frequency is the main contributor to the variance of F_{95} in all four regions under consideration. Plots a-b were created in MATLAB R2016a.

Supplementary Note 4: Local Perturbation Analysis

To gain additional insight on how small changes (as opposed to long-term climate change triggered changes) in intensity and frequency of storms impact the yearly affected population we perform a local perturbation analysis, where we evaluate the relative change in $\bar{F}$ and F_{95} with regards to changes in f_{fre}, f_{fre}^{sev}, and f_{int} in the vicinity of baseline scenario. Figure 4 reveals it is the change in f_{int} that most significantly impacts $\bar{F}$ and F_{95} suggesting that under the baseline scenario, the power system is more vulnerable toward increase in intensity of storms compared to increase in frequency of storms. It is also seen that, under baseline scenario, *relative* increase in frequency of non-intense storms impacts the power system more significantly than *relative* increase in frequency of intense storms.

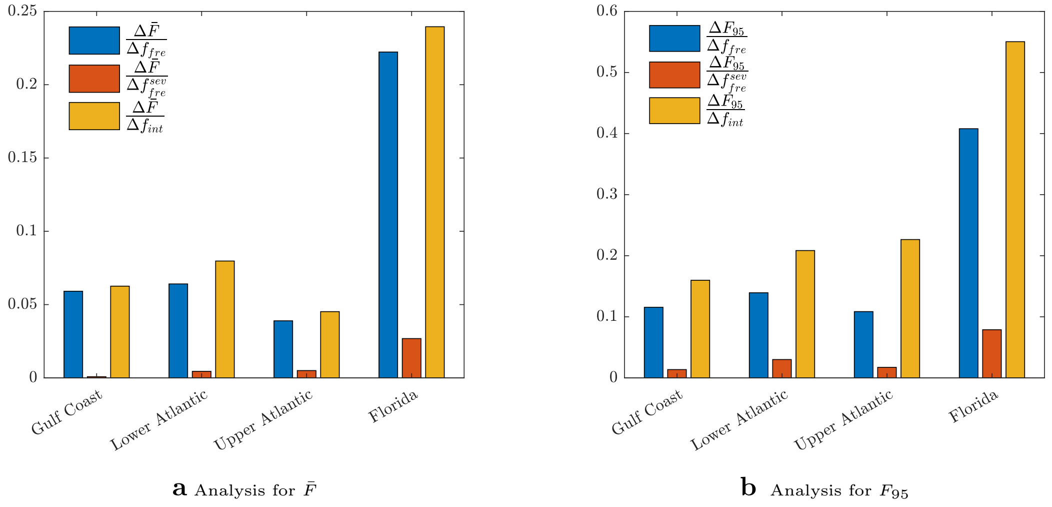

Figure 4: Local perturbation analysis. Figure shows relative changes in mean and 95th percentile of yearly fraction of customers that experience storm-induced power outages with regards to changes in f_{fre}, f_{fre}^{sev}, and f_{int} in the vicinity of baseline scenario. Plots a-b were created in MATLAB R2016a.
